# IRONMAP: Iron network mapping and analysis protocol for detecting over-time brain iron abnormalities in neurological disease

**DOI:** 10.1162/imag_a_00528

**Published:** 2025-04-15

**Authors:** Jack A. Reeves, Fahad Salman, Michael G. Dwyer, Niels Bergsland, Sarah Muldoon, Bianca Weinstock-Guttman, Robert Zivadinov, Ferdinand Schweser

**Affiliations:** Buffalo Neuroimaging Analysis Center, Department of Neurology, Jacobs School of Medicine and Biomedical Sciences, University at Buffalo, State University of New York, Buffalo, NY, United States; Center for Biomedical Imaging at the Clinical Translational Science Institute, University at Buffalo, State University of New York, Buffalo, NY, United States; Department of Mathematics, University at Buffalo, State University of New York, Buffalo, NY, United States; Institute for Artificial Intelligence and Data Science, State University of New York, Buffalo, NY, United States; Jacobs Neurological Institute, Buffalo, NY, United States

**Keywords:** brain iron, deep gray matter, multiple sclerosis, network neuroscience, neurological disorders, quantitative susceptibility mapping

## Abstract

Altered iron levels, detected using iron-sensitive MRI techniques such as quantitative susceptibility mapping (QSM), are observed in neurological disorders and may play a crucial role in disease pathophysiology. However, brain iron changes occur slowly, even in neurological diseases, and can be influenced by physiological or environmental factors that are difficult to quantify in the research or clinical settings. Therefore, novel analysis methods are needed to improve sensitivity to disease-related iron changes beyond conventional region-based approaches. This study introduces IRONMAP, Iron Network Mapping and Analysis Protocol, which is a novel network-based analysis method to evaluate over-time changes in magnetic susceptibility. With this technique, we analyzed short-term (<1 year) longitudinal QSM data from a cohort of people with multiple sclerosis (pwMS) and healthy controls (HCs) and assessed disease-related network patterns, comparing the new approach to a conventional per-region rate-of-change method. IRONMAP revealed over-time, MS-related brain iron abnormalities that were undetectable using the rate-of-change approach. IRONMAP was applicable at the per-subject level, improving binary classification of pwMS vs. HCs compared to rate-of-change data alone (areas under the curve: 0.773 vs. 0.636, p = 0.024). Further analysis revealed that the observed IRONMAP-derived HC network structure closely aligned with simulated networks based on healthy aging-related susceptibility data, suggesting that disruptions in normal aging-related iron changes may contribute to the network differences seen in pwMS. IRONMAP is applicable to various neurological diseases, including Alzheimer’s disease and Parkinson’s disease, and can be used between any set of brain regions. Our proposed technique may allow for the study of brain iron abnormalities over shorter timeframes than previously possible.

## 1 Introduction

Maintaining brain iron homeostasis is critical for healthy brain function (Singh et al., 2014). Adequate iron levels are necessary for normal metabolic processes, including myelination and neurotransmitter synthesis, but excessive (improperly sequestered) iron can lead to the formation of reactive oxygen species with harmful effects (Connor & Menzies, 1996;Dixon & Stockwell, 2014;Kuhn et al., 1980). Several factors have been linked to altered iron homeostasis in the brain, including healthy aging (Hallgren & Sourander, 1958), clinical factors such as body mass index (Li et al., 2021), and neurological disorders like Alzheimer’s disease and multiple sclerosis (MS) (Damulina et al., 2020;Stankiewicz et al., 2014). Various cellular and biochemical mechanisms have been proposed to contribute to these iron alterations, such as altered iron transport across the blood-brain barrier, inflammatory activity, or iron depletion from the glial syncytium leading to decreased concentration (Schweser et al., 2021;Stankiewicz et al., 2014).

Iron levels in the brain can be assessed non-invasively using iron-sensitive MRI techniques like quantitative susceptibility mapping (QSM) (Reichenbach et al., 2015;Schweser et al., 2011,^2016^), which is influenced by iron due to its strong paramagnetic properties (Langkammer et al., 2012). However, magnetic susceptibility is not exclusive to iron and can also be impacted by other factors such as myelin, calcium, and inflammation (Lambrecht et al., 2020). In the deep gray matter (DGM) structures, the dominant contribution to susceptibility changes has been attributed to iron content, as supported by histological studies correlating MRI-based susceptibility measurements with iron concentrations (Langkammer et al., 2010,^2012^;Ropele & Langkammer, 2017). Nevertheless, in the context of MS, demyelination and inflammatory processes may also affect QSM signals in a short period of time. Despite this complexity, previous studies suggest that QSM changes in DGM regions of people with MS are primarily driven by iron abnormalities (Schweser et al., 2021), which is further supported by the naturally lower levels of myelin in these structures compared to the white matter (Reichenbach et al., 2015).

In healthy individuals, MRI studies have demonstrated that aging-related deep gray matter (DGM) iron dynamics vary between regions. For example, iron levels in the globus pallidus rapidly increase in adolescence and plateau around age 30, whereas iron accumulates in the caudate at a slower pace throughout the human lifespan (Aquino et al., 2009;Zhang et al., 2018). Such MRI findings are consistent with post-mortem histochemical iron measurements (Hallgren & Sourander, 1958). These findings suggest that certain DGM regions, such as the caudate and hippocampus, which both slowly accumulate iron over time (Zhang et al., 2018), may have similar iron dynamics and may even share common iron transport mechanisms. This latter idea is supported by a recent study showing that brain iron can be directly translocated between brain regions via axons (Wang et al., 2019).

In people with MS (pwMS), MRI studies have shown increased iron concentrations in DGM regions such as the putamen and caudate, while showing decreased concentrations in the pulvinar of the thalamus (Hagemeier et al., 2018;Khalil et al., 2015;Voon et al., 2024;Walsh et al., 2014). DGM iron alterations detected on MRI predict clinical disability, disease subtype, and disease duration, independent of atrophy and white matter (WM) lesion load (Zivadinov et al., 2018). Additionally, recent work has shown a link between pulvinar iron decreases and paramagnetic rim lesions, an imaging marker of chronic white matter inflammation (Reeves et al., 2025). A limitation of observing over-time DGM iron alterations is that they change slowly and can be influenced by confounding physiological or environmental factors such as diet, which are difficult to quantify in the research or clinical settings (Hagemeier et al., 2015;Liu et al., 2016). Therefore, monitoring longitudinal iron changes requires relatively large cohorts and long follow-up times, even for group-level analyses (Hagemeier et al., 2018;Khalil et al., 2015). Novel analysis methods with improved sensitivity to over-time DGM iron abnormalities are needed for translation of DGM iron as a clinical neuroimaging marker.

We recently reported a network-based method that leveraged independent component analysis (ICA) to identify covarying patterns (“networks”) of susceptibility change in cross-sectional data of pwMS and healthy controls (HCs) (Reeves et al., 2022). The approach improved sensitivity in detecting MS-related magnetic susceptibility alterations compared to conventional per-region susceptibility analysis (Reeves et al., 2022). Additionally, several recent studies by other investigators applied network approaches to detect susceptibility alterations in people with schizophrenia (Chen et al., 2024;Ravanfar et al., 2022). Together, these studies show that network analyses may be useful in detecting disease-specific iron alterations (Chen et al., 2024;Ravanfar et al., 2022;Reeves et al., 2022), and may improve sensitivity as compared to the conventional method of evaluating susceptibility levels in each region separately (Reeves et al., 2022). Additionally, as shown byWang et al. (2019), comparing iron dynamics between regions may provide valuable insight into healthy brain iron physiology and disease pathophysiology. A limitation of these previous network-based approaches is that they were only applied to cross-sectional data. Additionally, comparisons were performed between subjects. Therefore, the utility of network-based analyses in detecting over-time iron alterations in individual subjects is unknown.

In the present work, we introduce IRONMAP, Iron Network Mapping and Analysis Protocol. IRONMAP is a novel network analysis approach for studying over-time magnetic susceptibility changes. We hypothesized that this new method exposes short-term (<1 year) disease-specific iron alterations in pwMS undetectable with conventional longitudinal per-region methods. We tested the hypothesis by analyzing QSM data from a cohort of pwMS and comparing the resulting IRONMAP-derived network patterns to those observed in HCs. We assessed whether values obtained from the IRONMAP approach improved classification of*individual*subjects as pwMS vs. HCs, as compared to per-region rates of susceptibility change alone. Finally, we investigated the potential physiological basis for the observed IRONMAP-derived HC network patterns. To do so, we used numerical simulations to test whether the*in vivo*HC network patterns were similar to those expected from normal aging-related iron changes.

## 2 Methods

### 2.1 Participants and data collection

This study included previously collected data identified in our imaging database of IRB-approved studies. Subjects were included if they had at least three MRI scans on the same 3T MRI scanner within one year with the same 3D gradient-echo sequences (GRE), and either had clinically definite MS (pwMS) or were neurologically normal (i.e., HCs). Subjects were excluded if at least one of the identified scans was deemed unusable due to excessive motion or other artifacts. Exactly three MRI scans per subject were included for analysis, with the most recent set of three (acquired within a one-year timeframe) being selected for analysis if the subject had additional sets of scans that fit the inclusion criteria.

Written informed consent was obtained from all participants according to the Declaration of Helsinki. Demographic and clinical data were collected during an in-person interview and with additional standardized questionnaires. Information on gadolinium administrations in pwMS was collected via retrospective evaluation of electronic medical records.

### 2.2 MRI acquisition, reconstruction, and conventional ROI analysis

We used a 3T GE Signa Excite HDx 23.0 MRI scanner (General Electric, Milwaukee, WI, USA), and the protocols included both a spoiled single-echo 3D GRE and high-resolution 3D T1-weighted (T1w) imaging. GRE imaging parameters were as follows: matrix size of 512 x 192 x 68 mm^3^, voxel size of 0.5 x 0.5 x 2mm³, flip angle of 12°, TE/TR of 22ms/40ms, bandwidth of 13.89 kHz, and acquisition time 8:46 min:sec. T1w imaging parameters were: FOV 256 x 192 x 128 mm^3^, nominal 1 mm^3^resolution (FOV = 256 × 192 × 192 mm^3^), TE/TI/TR=2.8ms/900ms/5.9 ms, and acquisition time 4:49 min:sec. The scanner did not undergo any major hardware or software upgrades throughout the study. Susceptibility maps were reconstructed from raw GRE k-space data using scalar phase matching (de Zwart et al., 2002;Hammond et al., 2008), path-based phase unwrapping (Abdul-Rahman et al., 2007), background field removal by solving the Laplacian boundary value (LBV) problem, and Superfast Dipole Inversion (SDI) (Zhou et al., 2014;Schweser et al., 2013). Susceptibility maps were then whole-brain referenced.

We segmented 10 DGM regions (left and right caudate, hippocampus, pallidum, putamen, and thalamus) for each individual subject and each timepoint separately. To do so, we first rigidly aligned the magnitude GRE images with their respective T1w images using FSL FLIRT with 6 degrees-of-freedom (DOF) and trilinear interpolation (default for resampling) (Jenkinson et al., 2002;Patenaude et al., 2011). We utilized T1w images as the initial target for the alignment due to their better-defined cortical contrast compared to GRE/QSM images. Following this, the inverse of the resulting transformation matrix was utilized to co-register T1w images to their respective susceptibility maps. Following this procedure, we applied FSL FIRST automated segmentation to the susceptibility map-registered T1w images (Patenaude et al., 2011). The segmented DGM labels were then carefully inspected on each subject’s corresponding susceptibility map by a neuroimaging researcher (J.A.R., with 4 years of experience) and manually corrected as needed. This approach offers greater precision than co-registering all scans and using a single segmentation, which, while better for maintaining regional boundaries across scans, risks susceptibility value corruption due to partial volume effects (Tohka, 2014). On the other hand, tailoring DGM segmentations to each scan, as in the current approach, introduces slight variations in regional boundaries across scans. Subsequently, the region of interest (ROI) volumes and mean susceptibility values (from the T1w-derived ROIs in native-space susceptibility maps) were calculated for each timepoint for each participant. A representative susceptibility map and its ROI segmentations are shown inFigure 1Aand1B, respectively.

**Fig. 1.**
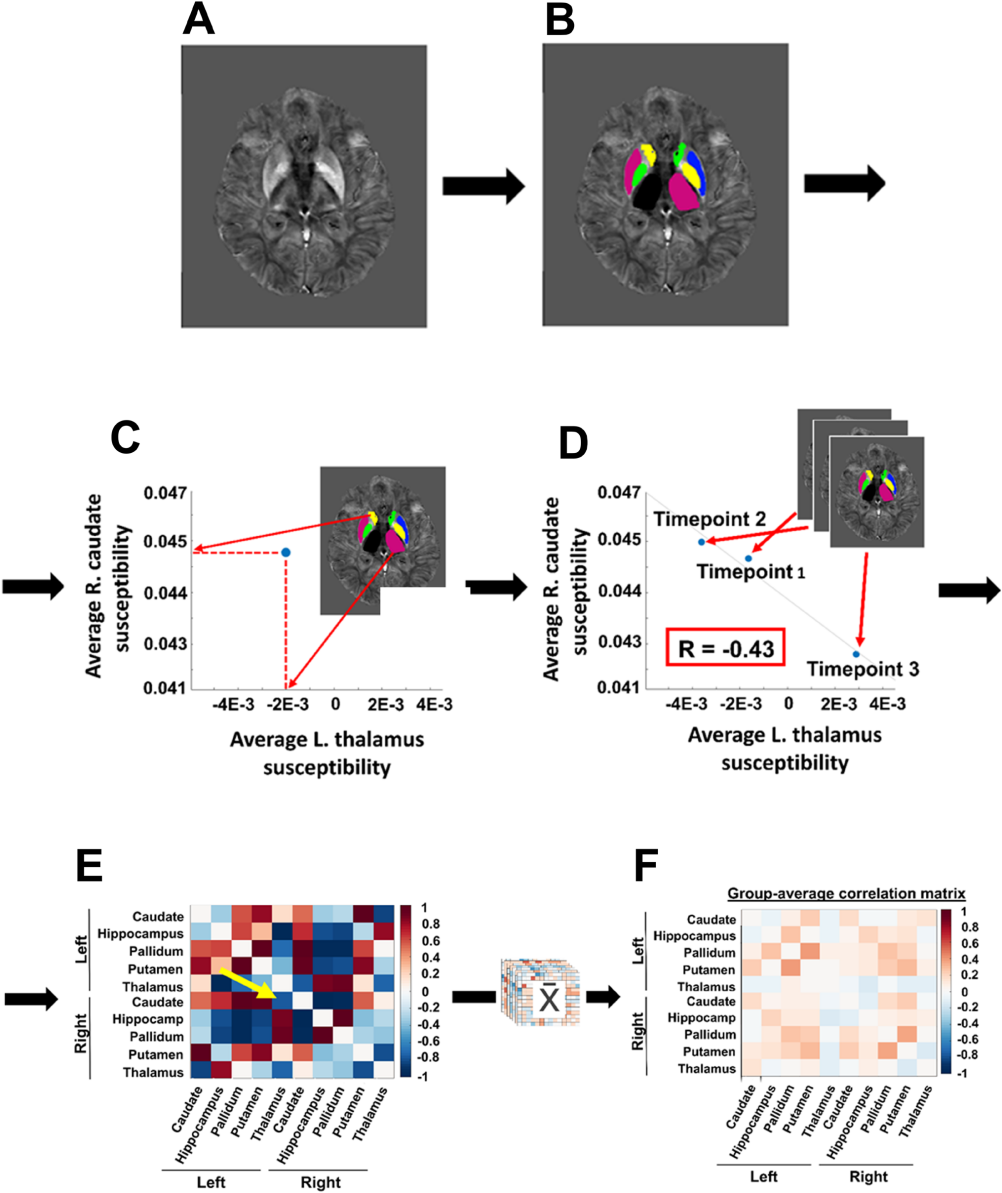
Procedure for the proposed network analysis. (A) Representative susceptibility map in native subject space. (B) Corresponding deep gray matter segmentation. (C) Graph illustrates the average susceptibility in the left thalamus compared to the right caudate at a single timepoint. (D) Pearson correlation between the left thalamus and right caudate across three timepoints of the same subject. (E) Correlation coefficients of all region pairs of the representative subject organized into a correlation matrix. The yellow arrow in (E) points to the correlation coefficient generated in (D). (F) Group-average correlation matrix.

### 2.3 IRONMAP methodology

The standard ROI-based approach can assess over-time changes in susceptibility in individual regions. Here, we generalize this approach toward assessing the relationship of over-time susceptibility changes*between*regions. Specifically, we described the temporal dynamics of the susceptibility as a weighted graph in which each node represents an anatomical region and the connections between the nodes, or “edges”, carry a weight that is defined by strength of the over-time correlation of the susceptibility between two regions. A positive correlation between two regions indicates that their susceptibilities tend to increase or decrease in tandem, whereas a negative correlation suggests that an increase in susceptibility in one region is associated with a decrease in the other. We calculated the 45 unique edge weights corresponding to region pairs generated by Pearson correlating the region-average susceptibility values of the two anatomical regions across timepoints, as illustrated inFigure 1C, and organized the values into individual-subject correlation matrices, as illustrated inFigure 1D. We then calculated group-average correlation matrices for pwMS and HCs separately, as illustrated inFigure 1F.

### 2.4 IRONMAP analysis of aging-related susceptibility networks (in silico)

We investigated if aging-related changes in susceptibility could explain the IRONMAP-derived in vivo network structure in HCs. We based these simulations on the aging trajectories of magnetic susceptibility previously published for five bilateral DGM structures (caudate, hippocampus, pallidum, putamen, and thalamus) (Zhang et al., 2018). We determined for each subject and timepoint the putative magnetic susceptibility values for each region using the age of the subject at the time of the scan. From these values, we determined the expected over-time change of the regional susceptibilities from the baseline to each follow-up timepoint. These changes were added to each subject’s observed baseline susceptibility to simulate short-term susceptibility values for each DGM region. We systematically investigated the effect of random noise by adding zero-mean Gaussian noise to the simulated susceptibility values with varying standard deviations from 0.01 ppb to 1.0 ppb in steps of 0.01 ppb. This process is illustrated inFigure 2.

**Fig. 2.**
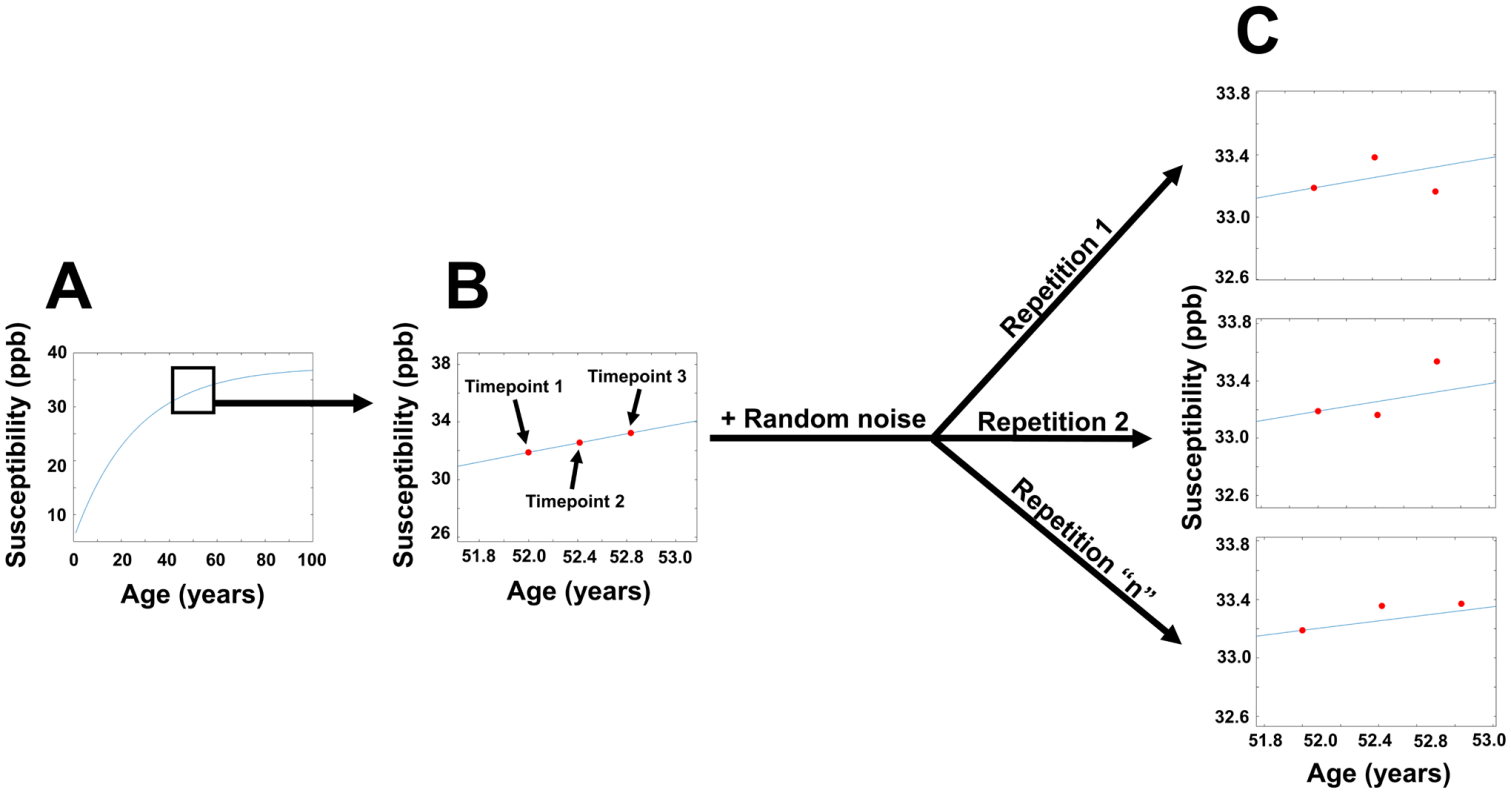
Procedure for generating simulated magnetic susceptibility data. (A) Putative susceptibility curve for sample region (left caudate). (B) Putative susceptibility values based on sample subject’s age at each timepoint. (C) Iterative addition of random Gaussian noise to the follow-up timepoints.

We then performed the IRONMAP analysis on the simulated data, generated weighted graphs for each subject, and compared the simulated graphs to the graphs observed in vivo. These last steps were repeated with simulated values generated using zero-mean Gaussian noise to determine the specificity of our comparisons for age-related changes, as opposed to random noise. The simulated aging analysis and noise-only analyses were repeated 1000 times each with randomly varying Gaussian noise.

### 2.5 Statistics

Statistical analyses were conducted using MATLAB (version 2019b; The MathWorks, Natick, MA) unless otherwise stated. Subject ages and time between first and third MRIs were compared between HC and pwMS groups using two-tailed independent-samples T-tests, and sex was compared using a chi-squared test. Statistical significance was considered at alpha < 0.05 for all analyses.

#### 2.5.1 Removing the confounding effects of volume and gadolinium accumulation

Prior to the network analysis, linear regression was used to regress out the effect of volume on mean susceptibility for each region (across all scans) and the resulting residual values were saved. Subsequently, to control for potential confounding effects of gadolinium accumulation from contrast agent injections in the patient group (Choi et al., 2020;Guo et al., 2018;Wattjes et al., 2021), per-region regression models were fit on the pwMS residuals using the number of gadolinium administrations since baseline scan as a predictor variable. The volume-corrected susceptibility residuals for the HC group and the volume- and gadolinium-corrected susceptibility residuals for the pwMS group were used in subsequent network analyses.

#### 2.5.2 Baseline and longitudinal DGM susceptibility comparisons

Baseline average susceptibilities were compared between pwMS and HCs using two-tailed independent-samples T-tests.

For longitudinal comparisons, the rate of susceptibility change in each region for each subject was calculated by fitting linear regression models with susceptibility residuals as outcome variables and per-visit subject ages as predictor variables. The rates of susceptibility change were the age beta coefficients (slopes) obtained from the regression. Beta coefficients were compared between pwMS and HCs using two-tailed independent-samples T-tests. Additionally, the rates of susceptibility change for each group (pwMS and HCs separately) were tested for non-zero change using two-tailed one-sample T-tests.

Note that this regression procedure was chosen over calculating pre-to-post changes (i.e., the difference between the first and last scans) in order to incorporate all data into the estimation of susceptibility changes, and to ensure that rate-of-change vs. network analyses comparisons used similar data (seeSections 2.5.4and3.4).

#### 2.5.3 Comparison of in vivo susceptibility network dynamics between PwMS and HCs

Prior to comparisons, each correlation coefficient (“r”) was transformed to a Z-score using the Fisher Z-transformation (i.e.,*Z*= arctanh(*r*)). Values of r = 1 and R = -r were set to 0.99 and -0.99 prior to the Z-transformation to avoid undefined values (i.e., infinity and negative infinity).

The number of numerically positive and negative Z-transformed correlation coefficients (of the 45 unique region-pairs) were compared between pwMS and HCs using chi-squared tests. The average difference in Z-scores was compared between pwMS and HCs by calculating the pwMS and HC Z-score averages for each unique region-pair, subtracting the HC averages from the pwMS averages, and performing a two-tailed one-sample T-test on the 45 mean-differences. This latter analysis was repeated using the absolute values of the Z-scores, to investigate whether observed differences in node strengths between groups were due to differences in correlation magnitude (e.g., correlation coefficients of 0.6 vs. 0.2) or differences in correlation sign (e.g., correlation coefficients of 0.2 vs. -0.2).

#### 2.5.4 Comparison of classification of pwMS and HCs using susceptibility rates vs. the IRONMAP approach

Binary regressions and receiver operating characteristic (ROC) analysis were used to test whether the correlations from the network approach added additional information for identifying MS-related pathology, as compared to only the rates of per-region susceptibility change. This analysis was performed using SPSS version 29.0 (IBM, Armonk, NY, United States).

An initial “Rate Only Model” was fit with subject group as the outcome variable and the 10 per-region (i.e., five DGM structures, left and right hemispheres) rates of susceptibility change added as forced entry predictor variables. A second “Rate + Network Model” was then fit. The “Rate + Network Model” used forward selection (at p < 0.05) to add only significant region-pair correlations to the model (from the 45 unique network region-pair correlations), along with the 10 per-region rates of susceptibility change as forced entry predictor variables. The predicted mean response of both final models was saved and used to generate ROC curves. Paired-sample area-under-the-curve (AUC) tests were then used to compare the ROC curve generated from the “Rate + Network Model” to the “Rate Only Model”. Additionally, Z-transformed correlation coefficients for the network region-pairs in the final “Rate + Network Model” were compared between pwMS and HCs using two-sided independent-samples T-tests.

#### 2.5.5 Comparison of in vivo and simulated (in silico) IRONMAP-derived networks

We quantified the similarity between the observed HC in vivo network and the simulated (in silico) HC aging networks by correlating their group-average matrix elements. We hypothesized that regions with similar aging-related iron dynamics, such as the hippocampus and caudate, would exhibit high in silico correlations. If aging-related iron changes were closely related to the in vivo network behavior, we expected region pairs with strong in silico correlations to also show strong in vivo correlations. Conversely, if aging-related iron changes had little relation with the in vivo network, we anticipated weak or no correlation between the in vivo and in silico networks.

For the in vivo network, each unique matrix element (n = 45) was averaged across HCs. For each noise level (n = 101) and each simulated network iteration (n = 1000), each unique in silico matrix element was averaged across HCs. Pearson correlations were then calculated between the in vivo and in silico matrix elements. For each noise level, the correlation coefficients were averaged across the n = 1000 iterations. This procedure was also applied to noise-only matrices to determine if the observed network patterns could be explained by correlated Gaussian noise.

## 3 Results

### 3.1 Demographic characteristics

The database search identified 99 pwMS (baseline age = 43.7 ± 11.2 years, 66.7% female) and 29 HCs (baseline age = 44.1 ± 15.6 years, 72.4% female) who met the inclusion criteria and were included in subsequent analyses. There were no significant differences between pwMS and HCs in baseline age (p = 0.764), sex (p = 0.654). The pwMS cohort had a slightly longer interval between first and third scans (0.7 ± 0.2 years) compared to the HC cohort (0.6 ± 0.2 years; p = 0.044). Details on age, sex, disease duration, and clinical disability as assessed by the Expanded Disability Status Scale (EDSS) are provided inTable 1.

**Table 1.** Demographic information for the people with MS and healthy controls.

Characteristic	pwMS	HCs	p-value
N	99 (85 pwRRMS, 14 pwSPMS)	29	
Age, yrs (mean ± SD)	43.7 ± 11.2	44.1 ± 15.6	0.764 ^a^
Time between first and third MRIs, yrs (mean ± SD)	0.7 ± 0.2	0.6 ± 0.2	0.044 ^a^
Sex, n			0.654 ^b^
Female	66	21	
Male	33	8	
dd, yrs (mean ± SD)	12.0 ± 8.8		
EDSS (median [IQR])	3.0 [1.5 – 4.5]		
Number of gadolinium administrations over study timeframe (mean ± SD)	1.6 ± 0.7		

dd: disease duration; EDSS: Expanded Disability Status Scale; pwMS: people with multiple sclerosis; HC: healthy control; pwRRMS: people with relapsing-remitting multiple sclerosis; pwSPMS: people with secondary progressive multiple sclerosis; SD: standard deviation; IQR: interquartile range.

a Two-tailed independent-samples T-test.

b Chi-squared test.

Of the 297 MRIs from pwMS, 248 were from observational studies, 35 were from prospective drug trials (26 interferon beta-1a, 5 glatiramer acetate, 3 fingolimod, and 1 teriflunomide), and 14 were from a prospective neurovascular surgery study. All HC MRI scans (n = 87) were acquired as non-disease controls from MS observational studies. All scans were acquired between 2008 and 2016.

### 3.2 Baseline and longitudinal DGM susceptibility comparisons

Details on mean DGM baseline susceptibilities and longitudinal susceptibility changes for pwMS and HCs are shown inTable 2. At baseline, pwMS had higher mean susceptibility in the left caudate (48.3 ± 14.6 ppb for pwMS and 41.3 ± 16.7 ppb for HCs, p = 0.032), right caudate (47.9 ± 15.8 ppb for pwMS and 39.2 ± 17.1 ppb for HCs, p = 0.011), left pallidum (115.5 ± 29.2 ppb for pwMS, 92.9 ± 28.1 ppb for HCs, p < 0.001), and right pallidum (107.6 ± 30.8 ppb for pwMS, 82.5 ± 29.8 ppb for HCs, p < 0.001). None of the DGM regions had significantly non-zero rates of susceptibility change in either the pwMS or HC group (p > 0.1), and all rates were similar between pwMS and HCs (p > 0.25).

**Table 2.** Baseline and longitudinal susceptibility levels in DGM regions.

		Baseline susceptibility (ppb)			Rate of susceptibility change (ppb/year)		
		pwMS	HCs	p-value *	pwMS	HCs	p-value *
Left	Caudate	48.3 ± 14.6	41.3 ± 16.7	**0.032**	1.3 ± 29.7	4.1 ± 19.2	0.639
	Hippocampus	6.2 ± 7.2	6.5 ± 8.1	0.870	1.4 ± 20.9	-1.1 ± 11.0	0.544
	Pallidum	115.5 ± 29.2	92.9 ± 28.1	**<0.001**	8.9 ± 80.1	-2.0 ± 25.2	0.473
	Putamen	59.8 ± 18.7	54.4 ± 23.9	0.201	2.5 ± 47.3	-2.3 ± 28.0	0.600
	Thalamus	4.5 ± 9.7	8.0 ± 6.8	0.071	-1.3 ± 18.0	-1.8 ± 16.5	0.894
Right	Caudate	47.9 ± 15.8	39.2 ± 17.1	**0.011**	0.9 ± 33.6	1.8 ± 20.2	0.891
	Hippocampus	6.0 ± 6.6	3.9 ± 7.0	0.149	0.9 ± 14.2	-0.4 ± 8.7	0.631
	Pallidum	107.6 ± 30.8	82.5 ± 29.8	**<0.001**	10.2 ± 70.2	-5.0 ± 28.9	0.260
	Putamen	59.6 ± 19.5	54.1 ± 23.0	0.206	2.0 ± 52.4	-5.9 ± 20.7	0.430
	Thalamus	3.5 ± 9.0	5.7 ± 6.0	0.236	-1.1 ± 18.2	1.3 ± 12.2	0.507

p-values < 0.05 are bolded.

DGM: deep gray matter, HCs: healthy controls; ppb: parts per billion; pwMS: persons with multiple sclerosis.

* Two-tailed independent-samples T-tests.

### 3.3 Comparison of in vivo susceptibility network dynamics between PwMS and HCs

The group-average pwMS IRONMAP-derived matrix for in vivo susceptibility correlations is shown inFigure 3A, the group-average HC matrix is shown inFigure 3B, and the pwMS-minus-HC subtraction matrix is shown inFigure 3C. Of the 45 unique region-pairs, the group-average correlation coefficients for pwMS were numerically negative in 14/45 (31.1%) pairs. In contrast, HCs had 6 out of 45 (13.3%) numerically negative pairs, which was significantly fewer than the pwMS (Chi-squared p-value = 0.043).

**Fig. 3.**
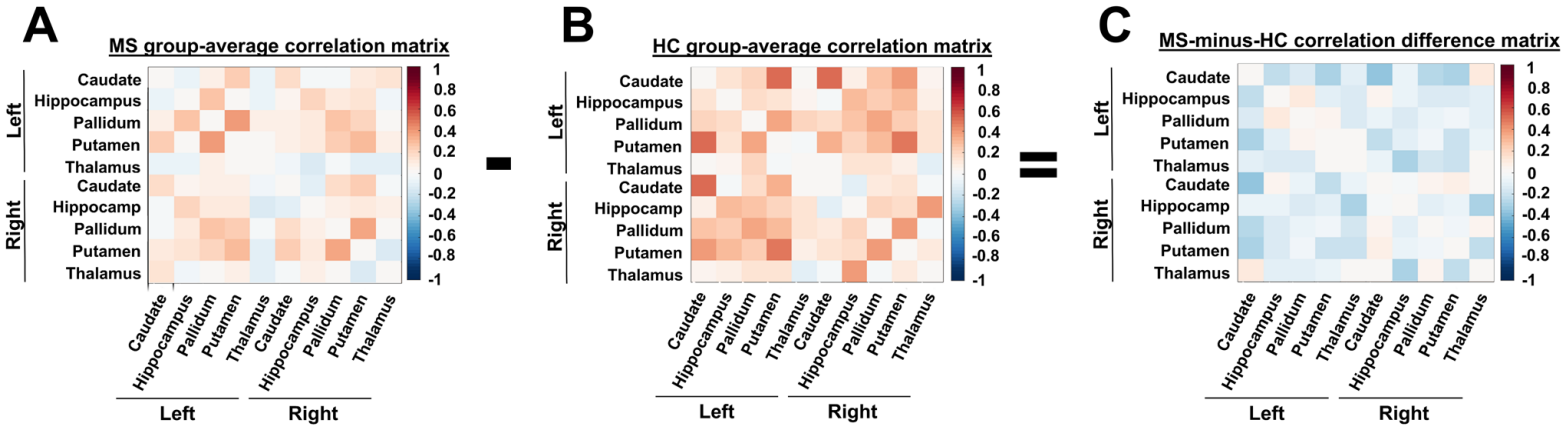
Group average correlation matrices for (A) people with MS, (B) healthy controls, and (C) difference between the matrices of people with MS and HC correlation matrix.

In the pwMS-minus-HC subtraction matrix, 35 (78.8%) of region-pairs were numerically negative. Across all unique region-pairs, average Z-transformed correlation coefficient values were lower in pwMS compared to HCs (mean for pwMS = 0.08 ± 0.13, mean for HCs = 0.18 ± 0.15, p < 0.001). Additionally, the average Z-transformed correlation coefficient magnitude was lower in pwMS compared to HCs (mean for pwMS = 0.66 ± 0.03, mean for HCs = 0.68 ± 0.05, p = 0.008).

### 3.4 Comparison of classification of pwMS and HCs using susceptibility rates vs. the IRONMAP approach

**Fig. 4.**
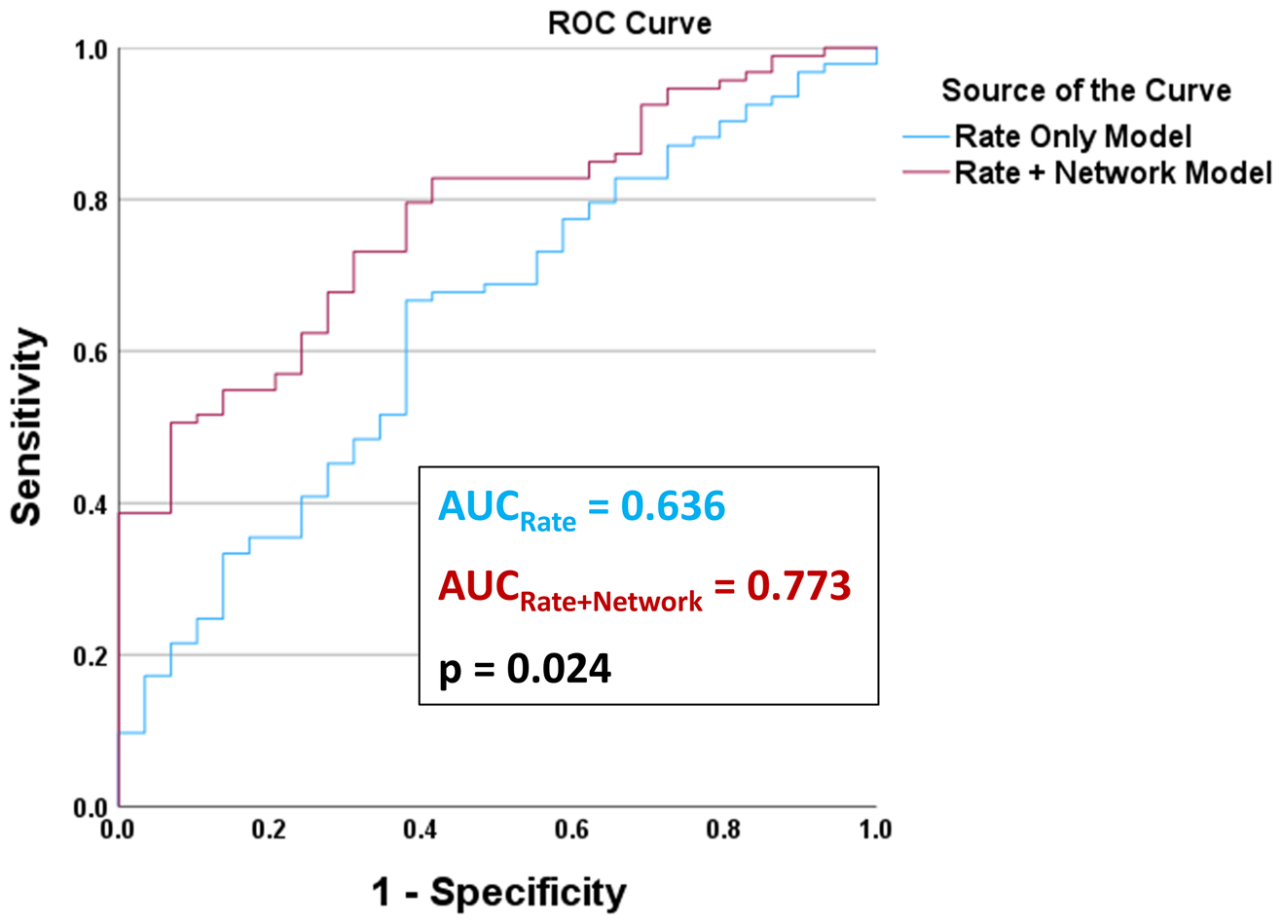
ROC curves for pwMS vs. HC classification using a “Rate Only Model” and a “Rate + Network Model”. Both models used binary logistic regression with subject group (pwMS or HC) as the outcome variable. The “Rate Only Model” included the 10 per-region (i.e., five bilateral DGM structures, left and right hemispheres) rates of susceptibility change as predictor variables. The “Rate + Network Model” used forward selection (at p < 0.05) to add only significant region-pair correlations to the model (from the 45 unique network region-pair correlations), with the 10 per-region rates of susceptibility change included as forced entry predictor variables. The final “Rate + Network Model” included correlation coefficients between the left caudate and left pallidum, the left caudate and right caudate, and the right pallidum and right thalamus.

Figure 4shows ROC curves for pwMS vs. HC classification using the Rate Only Model and for the final Rate + Network Model. The final Rate + Network Model included correlation coefficients between the left caudate and left pallidum, the left caudate and right caudate, and the right pallidum and right thalamus, in addition to the 10 per-region susceptibility rate changes. The AUC for the final Rate + Network Model (AUC = 0.773) was significantly greater than the AUC for the Rate Only Model (AUC = 0.636, p = 0.024).

Compared to HCs, pwMS had significantly smaller Z-transformed correlation coefficients between the left caudate and left pallidum (Z = 0.51 ± 0.57 for HCs and Z = 0.22 ± 0.68 for pwMS, p = 0.017), between the left caudate and right caudate (Z = 0.52 ± 0.48 for HCs and Z = 0.17 ± 0.74 for pwMS, p < 0.001), and between the right pallidum and right hippocampus (Z = 0.38 ± 0.59 for HCs and Z = 0.09 ± 0.71 for pwMS, p = 0.004).

### 3.5 Comparison of in vivo and simulated (in silico) IRONMAP-derived networks

**Fig. 5.**
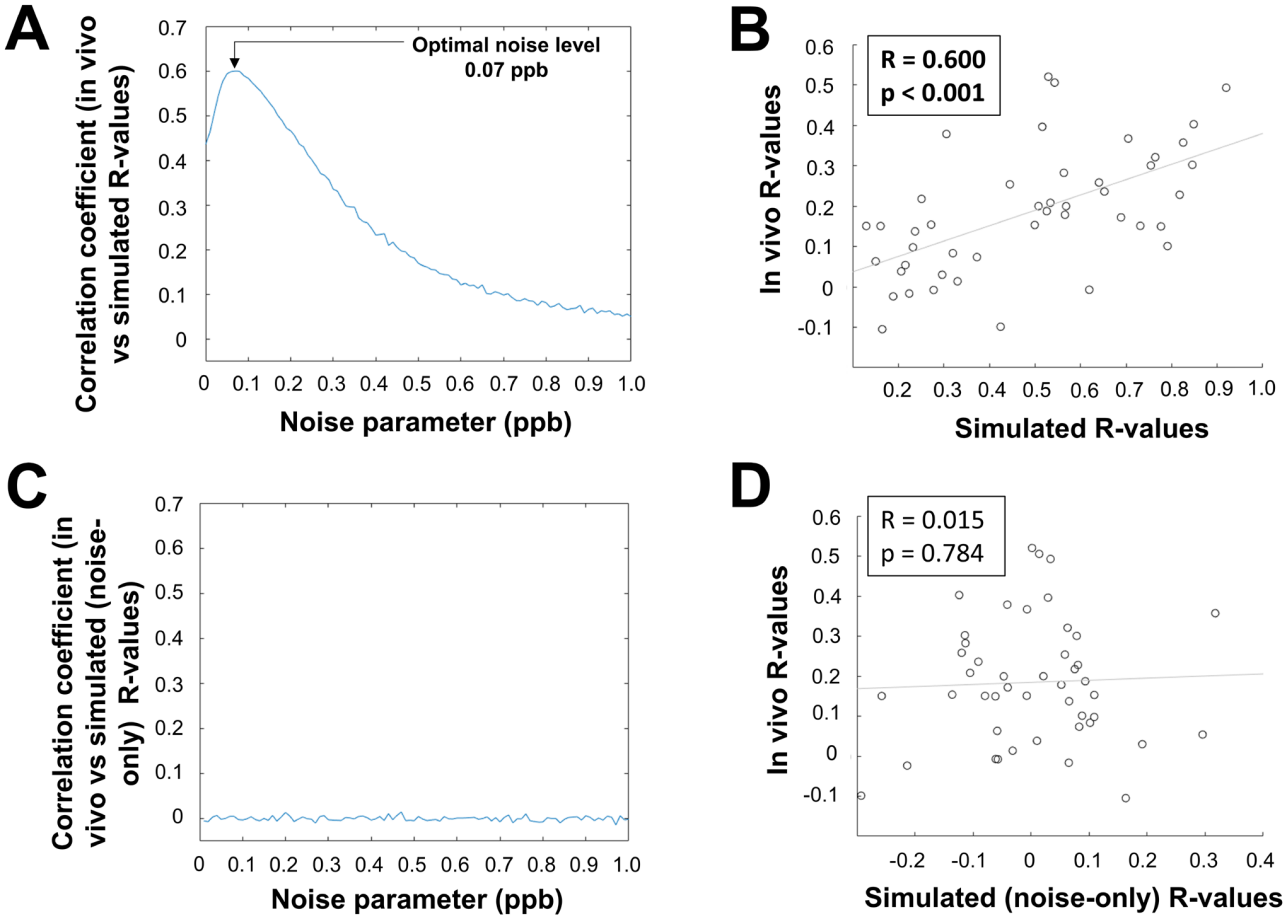
Association between simulated correlation coefficients and in vivo data. (A) Plot showing the relationship (correlation coefficients) between elements of the group-averaged HC in vivo network and corresponding elements of the simulated aging networks for different simulated noise levels, averaged over 1000 simulated iterations. (B) Scatter plot showing the group-averaged elements of the HC in vivo network (y-axis) and corresponding group-averaged elements of the simulated aging networks (x-axis) for the determined optimal noise level (0.07 ppb). (C) Plot showing the relationship (correlation coefficients) between elements of the group-averaged HC in vivo network and corresponding elements of the simulated noise-only networks for different simulated noise levels, averaged over 1000 simulated iterations. (D) Scatter plot showing the group-averaged elements of the HC in vivo network (y-axis) and corresponding group-averaged elements of the simulated noise-only networks (x-axis) for the determined optimal noise level (0.07 ppb).

Figure 5Acompares the unique group-average matrix elements of the HC in vivo network with corresponding elements of the simulated aging network for different noise levels. The correlation between in vivo and simulated values peaked at a noise level of 0.07 ppb with R = 0.600. At this noise level, the simulated aging-related susceptibility changes were able to explain R^2^= 36.0% of the observed temporal network dynamics. In contrast, the maximum absolute correlation observed with the noise-only simulation was R = 0.015 (Fig. 5C), equivalent to <0.1% of explained variance. Scatter plots showing the relationship between the unique matrix elements of the group-averaged HC in vivo network and the corresponding elements of the simulated aging network (noise level of 0.07 ppb) are shown inFigure 5B, and between the group-averaged HC in vivo network and noise-only simulated network inFigure 5D.

## 4 Discussion

In this study, we introduced IRONMAP, Iron Network Mapping and Analysis Protocol, which is a novel network analysis method for quantitative susceptibility mapping or other iron-sensitive metrics. We found that IRONMAP analysis improved detection of disease-specific susceptibility alterations (i.e., classification of pwMS vs. HCs) as compared to use of per-region rates-of-change. Comparison of our results to*in silico*numerical simulations showed substantial overlap between susceptibility network dynamics in HCs and network behavior expected by healthy aging. Together, these findings support IRONMAP as a sensitive tool for investigating brain iron dynamics in both healthy individuals and those with neurological diseases.

At baseline, we found higher susceptibility in the caudate and pallidum in pwMS compared to HCs. However, we did not detect significant over-time change susceptibility in either the pwMS or HC group in any DGM region, nor any rate differences between the groups. These findings are in line with previous studies showing that progressive brain iron changes occur slowly (Aquino et al., 2009;Hagemeier et al., 2018;Khalil et al., 2015). Therefore, although QSM provides a highly accurate measurement of magnetic susceptibility (Naji et al., 2022), measurement of progressive small over-time changes may be confounded by physiologic fluctuations in brain iron, that is, slight variations due to diet and lifestyle factors (Hagemeier et al., 2015;Liu et al., 2016). Fluctuations may influence the DGM itself or impact other brain regions used for QSM referencing (in our case, whole-brain referencing). In contrast, IRONMAP does not rely on progressive brain iron changes but instead on relative changes between brain regions. This feature allowed us to detect susceptibility alterations in pwMS compared to HCs in the caudate, hippocampus, pallidum, and thalamus, even in the absence of any detectable over-time changes in the individual regions. Another advantage of IRONMAP is that it evaluates correlations in ROI-averaged susceptibility values, rather than working on a voxel-by-voxel basis like our previous-described ICA method. This makes IRONMAP more robust to noise from MR acquisition and QSM reconstruction.

We found that between-region correlations generally decreased in magnitude in pwMS compared to HCs, and that there was a higher number of numerically negative correlations in pwMS. The Z-transformed correlation coefficients between the susceptibilities of the caudate, pallidum, and thalamus enhanced the ability to classify subjects as either pwMS or HCs. The magnetic susceptibility in each of these regions correlates with greater disability in pwMS (Zivadinov et al., 2018), further emphasizing the critical role of their iron dynamics in MS pathophysiology. Future studies may provide further insight into MS-related pathophysiology by evaluating the effects of disease-relevant factors (e.g., disease-modifying therapy use) on the IRONMAP network structure.

The group HC correlation matrix showed substantial agreement with correlation matrices generated from simulated aging data, with the simulated data explaining 36% of the observed in-vivo temporal network dynamics. Specifically, DGM regions that were predicted to be highly correlated due to similar aging-related brain iron dynamics, that is, caudate and the hippocampus, also had higher observed correlations in the*in vivo*data, and regions with dissimilar aging iron dynamics had weaker*in vivo*correlations. This finding suggests that the mechanisms driving similar long-term iron accumulation trajectories between the two regions may also underlie comparable short-term homeostatic iron dynamics, such as transient increases or decreases. In contrast, the noise-only simulations did not show any relationship with the in vivo data, indicating that random noise did not explain the*in vivo*findings. These findings indicate that much of the observed deviation seen in the pwMS network may be due to a breakdown of normal aging-related brain iron dynamics, rather than an artifact of correlated inter-scan variability/noise.

In a previous mouse study,Wang et al. (2019)showed that iron levels are negatively related between certain pairs of DGM regions, that is, iron chelation in the thalamus leads to*increased*iron levels in the substantia nigra. The authors interpreted this as certain areas providing “negative feedback” to other brain regions, possibly as a mechanism to maintain healthy brain iron homeostasis. In our data, we found an increased number of numerically negative between-region correlations in pwMS compared to HCs. Together, these results may indicate that pathological brain iron changes in different brain regions may directly interact, rather than occurring independently. If true, this may help explain why some brain regions (e.g., caudate) have increased brain iron in MS while other regions (e.g., pulvinar) have decreased iron. Future studies analyzing brain iron feedback on animal models, such as experimental autoimmune encephalomyelitis in mice, would provide additional information on this topic.

IRONMAP classification of subjects as pwMS and HCs (i.e., at the per-subject level) performed better as compared to conventional per-region methodology. It should be noted that our approach was not specifically optimized for detecting pwMS vs. HC differences. It is, therefore, likely that our approach could achieve greater group separation through improvements such as finer gray matter segmentation (e.g., thalamic subnuclei), increasing the number of MRI scans included in the network analysis, and including non-DGM regions, for example cortical regions, which are also known to have altered iron levels in neurological disease (Ravanfar et al., 2022). Optimized selection of disease-relevant brain regions may be particularly important for improving group selection because only a subset of the region-pairs were included in the final regression model, as reflected by the numerically similar (although statistically different) overall correlation coefficient averages for pwMS and HCs (i.e., 0.66 vs. 0.68). Although the susceptibility correlation methodology was applied to pwMS in the current study, it could also be applied to study other diseases with brain iron abnormalities, such as Parkinson’s disease and Alzheimer’s disease (Ravanfar et al., 2021).

## 5 Limitations

One limitation in interpretation of our results is that QSM signals are affected by both paramagnetic iron, which increases susceptibility relative to water, and diamagnetic myelin, which reduces susceptibility (Hametner et al., 2018;Langkammer et al., 2012). This is particularly relevant in our pwMS cohort, because both iron and myelin levels may be altered in neurodegenerative diseases. Therefore, our results need to be confirmed in future pathohistological studies, that is, in animal models. Alternatively, future human studies could incorporate QSM pre-processing techniques, such as chi-separation (Shin et al., 2021), to estimate the effects of iron and myelin separately on susceptibility levels.

Another confounding factor is atrophy, which may lead to increased iron concentrations if the same amount of iron remains in a smaller, atrophied region (Schweser et al., 2021). Additionally, although not intrinsic to the brain, administration of gadolinium as an MRI contrast has also been shown to affect QSM signal (Choi et al., 2020). Gadolinium is regularly administered in pwMS to evaluate the presence of acute lesions and has been shown to accumulate in brain tissue (Guo et al., 2018;Wattjes et al., 2021). We controlled for these potential effects by regressing out regional brain volumes (on the whole cohort) and the number of gadolinium administrations over the study interval (in pwMS). Despite this approach, it is still possible that gadolinium accumulation may have influenced our results. Specifically, gadolinium accumulation may have skewed the susceptibility averages of the pwMS cohort. Beyond gadolinium, DGM lesions, which we did not assess, may have influenced the measured susceptibility. Future studies should explore DGM lesions as a potential mechanistic factor influencing our findings.

Finally, the current study included subjects with three MRI scans within one year. This leads to two primary issues, the first being that the low number of timepoints used for correlations (i.e., 3) could cause our current analysis to be sensitive to biases in the data. Secondly, many pwMS and HCs in our local database had MRI scans acquired at yearly (or less frequent) intervals, leading to a relatively small number of HCs who matched this inclusion criterion. Moreover, although most MRIs from pwMS were conducted in observational studies (248/297 = 83.5%), the frequent scanning raises concerns about the generalizability of the findings in the present study to the broader MS population. Future prospective replications of our results are needed to better understand how short-term susceptibility dynamics relate to disease progression.

## 6 Conclusion

Our novel network-based analysis technique, IRONMAP, uncovered short-term, disease-related magnetic susceptibility abnormalities that were undetectable using a conventional per-region rate-of-change approach. IRONMAP has potential applications for studying longitudinal brain iron abnormalities in various neurological diseases, including MS, Alzheimer’s disease, and Parkinson’s disease, over shorter timeframes than previously feasible.

## Data Availability

The data and code used to generate the final results are available upon reasonable request to the corresponding author.
